# Transcaval versus Supra-Aortic Vascular Accesses for Transcatheter Aortic Valve Replacement: A Systematic Review with Meta-Analysis

**DOI:** 10.3390/jcm13020455

**Published:** 2024-01-14

**Authors:** Panagiotis Antiochos, Matthias Kirsch, Pierre Monney, Georgios Tzimas, David Meier, Stephane Fournier, Clémence Ferlay, Anna Nowacka, Valentina Rancati, Christophe Abellan, Ioannis Skalidis, Olivier Muller, Henri Lu

**Affiliations:** 1 Division of Cardiology, Lausanne University Hospital, University of Lausanne, 1011 Lausanne, Switzerland; panagiotis.antiochos@chuv.ch (P.A.); pierre.monney@chuv.ch (P.M.); georgios.tzimas@chuv.ch (G.T.); david.meier@chuv.ch (D.M.); stephane.fournier@chuv.ch (S.F.); clemence.ferlay@chuv.ch (C.F.); ioannis.skalidis@chuv.ch (I.S.); olivier.muller@chuv.ch (O.M.); 2 Division of Cardiovascular Surgery, Lausanne University Hospital, University of Lausanne, 1011 Lausanne, Switzerland; matthias.kirsch@chuv.ch (M.K.); anna.nowacka@chuv.ch (A.N.); 3 Adult Intensive Care Unit, Lausanne University Hospital, University of Lausanne, 1011 Lausanne, Switzerland; 4 Division of Anesthesiology, Lausanne University Hospital, University of Lausanne, 1011 Lausanne, Switzerland; valentina.rancati@chuv.ch; 5 Division of Internal Medicine, Lausanne University Hospital, University of Lausanne, 1011 Lausanne, Switzerland; christophe.abellan@chuv.ch

**Keywords:** transcaval, supra-aortic, transcatheter aortic valve replacement, TAVR, TAVI, meta-analysis

## Abstract

A growing body of evidence suggests that extrathoracic vascular accesses for transcatheter aortic valve replacement (TAVR) yield favorable outcomes and can be considered as primary alternatives when the gold-standard transfemoral access is contraindicated. Data comparing the transcaval (TCv) to supra-aortic (SAo) approaches (transcarotid, transsubclavian, and transaxillary) for TAVR are lacking. We aimed to compare the outcomes and safety of TCv and SAo accesses for TAVR as alternatives to transfemoral TAVR. A systematic review with meta-analysis was performed by searching PubMed/MEDLINE and EMBASE databases for all articles comparing TCv-TAVR against SAo-TAVR published until September 2023. Outcomes included in-hospital or 30-day all-cause mortality (ACM) and postoperative complications. A total of three studies with 318 TCv-TAVR and 179 SAo-TAVR patients were included. No statistically significant difference was found regarding in-hospital or 30-day ACM (relative risk [RR] 1.04, 95% confidence interval [CI] 0.47–2.34, *p* = 0.91), major bleeding, the need for blood transfusions, major vascular complications, and acute kidney injury. TCv-TAVR was associated with a non-statistically significant lower rate of neurovascular complications (RR 0.39, 95%CI 0.14–1.09, *p* = 0.07). These results suggest that both approaches may be considered as first-line alternatives to transfemoral TAVR, depending on local expertise and patients’ anatomy. Additional data from long-term cohort studies are needed.

## 1. Introduction

Although transfemoral (TF) vascular access is the gold-standard approach for transcatheter aortic valve replacement (TAVR), it may not be suitable in approximately 5–10% of patients due to anatomical challenges such as inadequate vessel size, severe calcification of the iliofemoral vasculature, or significant vessel tortuosity [1,2]. To circumvent these limitations, alternative vascular access routes have been developed, including extrathoracic (ET) approaches, which have shown improved outcomes compared to intrathoracic options. ET accesses can be further categorized into transcaval (TCv) [3] and supra-aortic (SAo) approaches, which include transcarotid (TC) [4], transsubclavian (TSc) [5], and transaxillary (TAx) [6] accesses. This classification is relevant because SAo approaches present broadly similar outcomes and prognoses, in addition to sharing common surgical characteristics [7]. As such, SAo approaches are now considered by many teams as the primary alternatives to TF access [7,8]. The TCv access, which involves percutaneously entering the abdominal aorta from the adjacent inferior vena cava, is the most recently developed approach [3]. Recent studies have demonstrated favorable outcomes associated with this access, leading some teams to advocate for its use as a first-line alternative to TF-TAVR [9]. Nonetheless, there is a scarcity of data comparing the TCv and SAo approaches. 

To our knowledge, no meta-analysis has directly compared TCv-TAVR to SAo-TAVR. Such an analysis would help define the best alternative to gold-standard TF access and is particularly relevant because there is no consensus defining the most appropriate approach when TF-TAVR is contraindicated. Our objectives were to compare the risks of in-hospital or 30-day all-cause mortality (ACM) and postoperative complications between TCv-TAVR and SAo-TAVR, among patients unfit for TF-TAVR.

## 2. Materials and Methods

This systematic review with meta-analysis adhered to a predetermined research plan, which was registered on the PROSPERO international prospective register of systematic reviews under the title “Transcaval Versus Supra-aortic Vascular Accesses for Transcatheter Aortic Valve Replacement: A Systematic Review with Meta-analysis; CRD42023439459”. The recommendations of the Committee of Medical Journal Editors (ICMJE) were followed in conducting this study [10]. The outcomes were reported in accordance with the guidelines outlined in the Preferred Reporting Items for Systematic Reviews and Meta-Analyses (PRISMA) statement [11]. Since this analysis relied on previously published data at the study level, ethical approval was not necessary.

### 2.1. Search Strategy and Selection Criteria

We conducted a systematic review encompassing all relevant data from inception until 26 September 2023, using the online databases PubMed/MEDLINE (Medical Literature Analysis and Retrieval System Online) and EMBASE (Excerpta Medica Database). The following keywords were used: “transcatheter aortic valve replacement,” “transcatheter aortic valve implantation,” “TAVR,” “TAVI,” “transcaval,” “caval-aortic,” “transcarotid,” “carotid,” “brachiocephalic,” “transsubclavian,” “subclavian,” “transaxillary,” and “axillary.” The study selection process is illustrated in Supplementary Table S1.

Initially, all references were included in the preliminary overview without any filters or restrictions. The studies selected for inclusion in this meta-analysis had to fulfill the following criteria: (1) they were original research articles; (2) they compared the TCv access against one or several SAo accesses; and (3) they reported patients’ baseline characteristics, 30-day ACM, and postoperative outcomes. Abstracts, poster presentations, case series, review articles, non-English language articles, and non-human studies were excluded.

The literature search, selection process, and data extraction were independently carried out by two co-authors (PA and HL). Any disagreement was resolved with the assistance of a third author (GT).

### 2.2. Outcomes

Outcomes included in-hospital or 30-day ACM (the two periods were grouped together) and immediate or 30-day postoperative complications (major bleeding and blood transfusions, major vascular complications, stroke or transient ischemic attack [TIA], acute kidney injury [AKI]). Whenever possible, outcomes were defined according to the Valve Academic Research Consortium-2 (VARC-2) or VARC-3 criteria [12,13].

### 2.3. Statistical Analyses

Continuous variables were presented as means with standard deviations (SDs) or medians with interquartile ranges (IQRs), while frequencies were used for dichotomous variables. Baseline characteristics were compared between groups using Pearson’s X^2^ test for categorical variables and Student’s *t*-test for normally distributed continuous variables. Data expressed as medians were not considered in the comparison statistical analyses as they were assumed not to follow a normal distribution. Relative risks (RRs) along with their corresponding 95% confidence intervals (CIs) were utilized as summary statistics and estimated using the Mantel-Haenszel random-effects model. We opted for this model to accommodate methodological variations and population diversity across the included studies. All *p*-values were two-sided, and a value below 0.05 was considered statistically significant. The percentage of total variation across studies due to heterogeneity rather than chance was estimated using the *I*^2^ statistic. Heterogeneity was categorized as low, moderate, or high based on intervals of <25%, 25–50%, and >50%, respectively. The quality assessment of observational studies was conducted using the Newcastle-Ottawa tool [14]. Statistical analyses were performed using the Review Manager 5.4 software (The Nordic Cochrane Center, The Cochrane Collaboration, Copenhagen, Denmark).

## 3. Results

### 3.1. Study Selection

**Figure 1:**
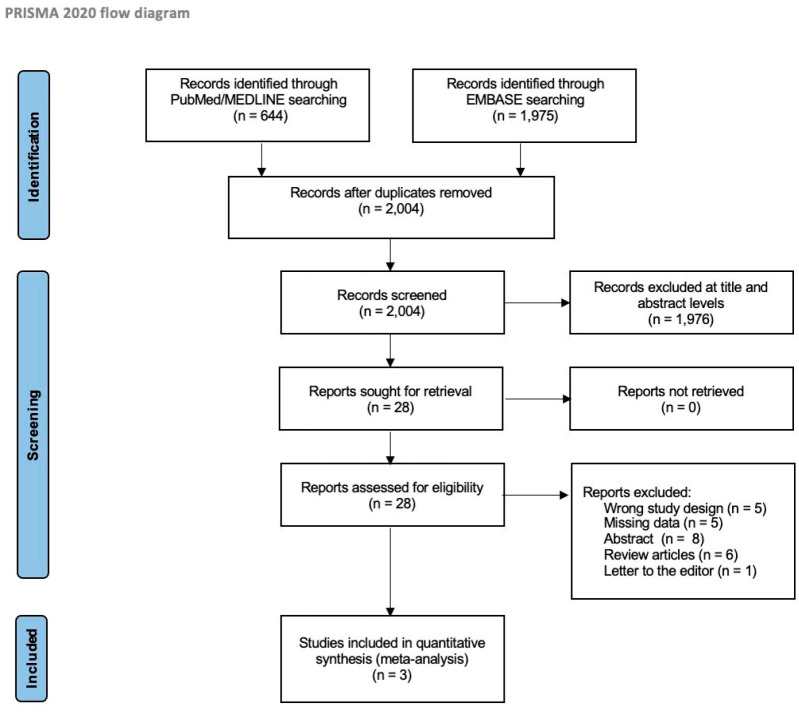
PRISMA Flowchart. PRISMA = Preferred Reporting Items for Systematic Reviews and Meta-Analyses.

A total of 2619 references were identified in the initial search. After removing duplicates, 2004 references remained. Of those, 1976 studies were excluded at the title and abstract levels. The remaining 28 studies were assessed for eligibility at the full-text level, with an additional 25 being excluded. After conducting a quality assessment of the three remaining studies using the Newcastle–Ottawa tool, we determined that two of them were of medium quality [15,16], while one study was of high quality [9]. All three studies met the criteria for inclusion in our meta-analysis (Supplementary Table S1). The PRISMA diagram illustrates the search strategy (Figure 1).

### 3.2. Patients’ Baseline Characteristics

The three studies included a total of 497 patients (318 in the TCv group and 179 in the SAo group). Their baseline clinical and echocardiographic characteristics are shown in Table 1. Among SAo patients, the TC access was used in 32 patients (17.9%), the TSc access in 41 patients (22.9%), and the TAx access in 106 patients (59.2%). The comparisons between TCv-TAVR and SAo-TAVR patients are presented in Supplementary Table S2 (categorical variables) and Supplementary Table S3 (continuous variables). TCv-TAVR patients presented more often with prior coronary artery bypass grafting (CABG, 25.8% vs. 17.0%, *p* = 0.04). Among patients in the TCv group, there was also a higher prevalence of peripheral artery disease, which did not reach statistical significance (65.0% vs. 50.7%, *p* = 0.07), as well as a lower proportion of men (43.3% vs. 52.0%, *p* = 0.07). Finally, TCv-TAVR patients were significantly younger than SAo-TAVR ones (77.3 ± 9.0 vs. 78.9 ± 8.4 years, *p* = 0.04). No statistically significant difference was found regarding the other baseline comorbidities or surgical risk score.

In two studies, the procedure selection process was reported [15,16]. In both cases, the TCv and SAo accesses were considered as first-line alternatives to TF-TAVR. The final decision between the two approaches was made collaboratively by the Heart Team, taking into account clinical data and the evaluation of the computed tomography scans.

### 3.3. Perioperative Characteristics

Data on perioperative characteristics are presented in Supplementary Tables S4 and S5. The proportion of patients receiving moderate sedation was significantly higher in the TCv-TAVR group (46.9% vs. 12.8%, *p* < 0.001). Procedural success was high and similar in the two groups (98.8% vs. 99.3%, *p* = 0.64), and no significant difference was found regarding the type of transcatheter heart valve that was utilized.

### 3.4. In-Hospital or 30-Day All-Cause Mortality

**Figure 2:**
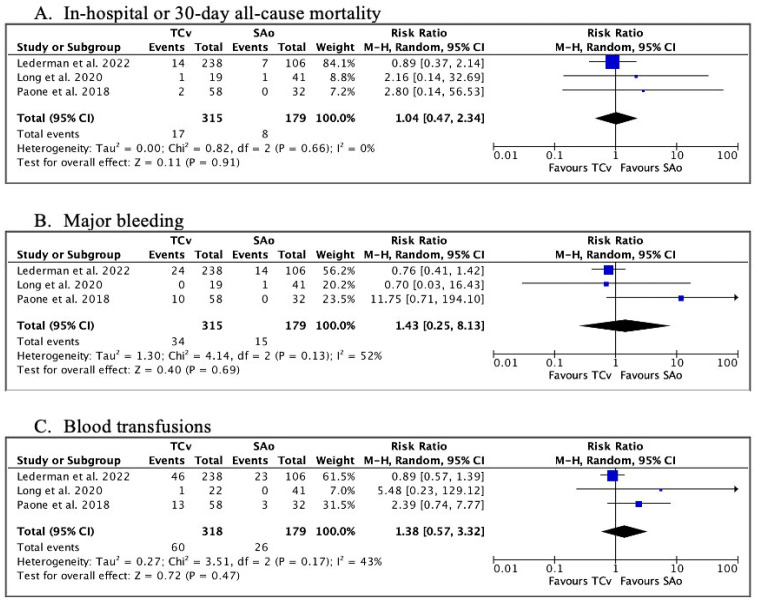
Forest plots. (**A**) Risk ratios for in-hospital or 30-day all-cause mortality, (**B**) Risk ratios for post-operative major bleeding, (**C**) Risk ratios for the need for blood transfusions [9,15,16].

Data regarding in-hospital or 30-day ACM were reported in all three studies. No significant difference was found between TCv-TAVR and SAo TAVR (RR 1.04, 95% CI 0.47–2.34, *p* = 0.91) (Figure 2A).

### 3.5. Major Bleeding and Blood Transfusions

Data on major bleeding were available in all three studies, with no significant difference found between the two groups (RR 1.43, 95%CI 0.25–8.13, *p* = 0.69) (Figure 2B). Likewise, there was no significant difference regarding the need for blood transfusions (RR 1.38, 95%CI 0.57–3.32, *p* = 0.17) (Figure 2C).

### 3.6. Major Vascular Complications

**Figure 3:**
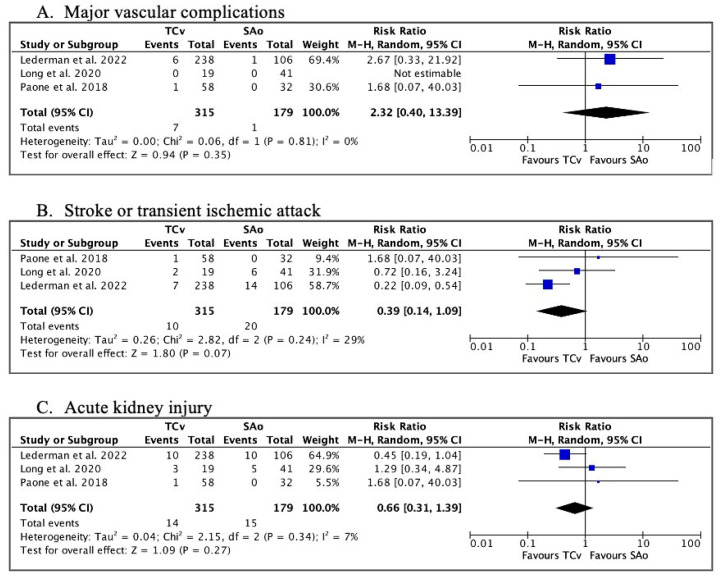
Forest plots. (**A**) Risk ratios for post-operative major vascular complications, (**B**) Risk ratios for stroke or TIA, (**C**) Risk ratios for the development of acute kidney injury. TIA = transient ischemic attack [9,15,16].

Data on major vascular complications were available in all three studies, with no significant difference found between TCv-TAVR and SAo-TAVR (RR 2.32, 95%CI 0.40–13.39, *p* = 0.35) (Figure 3A).

### 3.7. Stroke or Transient Ischemic Attack

Data regarding new-onset stroke or TIA within 30 days were found in all three studies. The TCv approach was associated with lower risk but did not reach statistical significance by a small margin (RR 0.39, 95%CI, 0.14–1.09, *p* = 0.07) (Figure 3B).

### 3.8. Acute Kidney Injury

Data regarding AKI were available in all three studies, with no significant difference between the two groups (RR 0.66, 95%CI 0.31–1.39, *p* = 0.27) (Figure 3C).

## 4. Discussion

To our knowledge, this meta-analysis is the first to compare the TCv vascular access against other ET accesses. Our main findings can be summarized as follows: (1) No statistically significant difference was found regarding in-hospital or 30-day ACM, major bleeding, the need for blood transfusions, major vascular complications, or acute kidney injury; (2) the TCv approach was associated with a lower rate of neurovascular complications, which however did not reach statistical significance; and (3) the proportion of patients receiving moderate sedation was higher in the TCv-TAVR group.

During the past decade, in patients unfit for the gold-standard TF vascular approach, SAo-TAVR has become the de facto first-line alternative for many teams worldwide. In a previously published meta-analysis, our team showed that SAo approaches, compared with the transthoracic ones (transapical and transaortic), were associated with lower ACM (30-day or 1-year) and lower rates of post-operative complications (including life-threatening bleeding, new-onset atrial fibrillation or flutter, and AKI needing renal replacement therapy), provided patient groups were not comparable (transthoracic-TAVR patients being more poly-morbid) [17]. Historically, transthoracic approaches were developed first and considered as the primary alternatives to TF access until the mid-2010s. They offer the advantage of easy access to the aortic valve; however, they are usually performed under general anesthesia and require a surgical cutdown of the thoracic wall, with the need for longer postoperative recovery periods. Observational data suggested that transthoracic approaches yield a higher 30-day ACM rate compared with the TF approach [18,19]. As such, the use of transthoracic-TAVR gradually decreased over time, while the use of SAo-TAVR, showing promising results (and in some cases, comparability to TF-TAVR [7,8]), witnessed an increase.

The TCv approach represents the latest major alternative vascular pathway developed for TAVR. Initial significant outcomes were first reported by Greenbaum and colleagues [20]. In a cohort of 100 high-surgical-risk patients (with a mean Euroscore II predicted risk of mortality of 10.9 ± 9.8%), the 30-day all-cause mortality rate stood at 8%. Additionally, rates of ischemic stroke, life-threatening bleeding, and major vascular complications were reported at 5%, 7%, and 13%, respectively. These encouraging findings have prompted certain teams to advocate for its consideration as a primary alternative to TF-TAVR [9].

A specific concern associated with TCv-TAVR is the potential for increased retroperitoneal bleeding complications, a phenomenon not observed in our study when compared to SAo-TAVR (no significant difference regarding the rate of life-threatening bleeding or the need for blood transfusion). In fact, this perceived increased risk is unfounded due to physiological factors. The TCv access relies on the principle that interstitial hydrostatic pressure surpasses venous pressure. Consequently, in the relatively restricted retroperitoneal space, blood that exits an opening in the abdominal aorta returns to the vena cava, preventing its accumulation as a hemorrhage in the retroperitoneal cavity [21]. These particular anatomical and physiological characteristics allow for a 2-step closure of aorto-caval tracts using permeable nitinol occluder devices after removal of the delivery system [20].

An important observation in our study pertains to the higher proportion of TCv-TAVR patients who underwent interventions under sedation with local anesthesia (almost half of them) compared to SAo-TAVR patients. While the use of local anesthesia with sedation has also been described in the latter group, previous data indicate that most teams prefer using general anesthesia [22]. This represents a clear advantage of the TCv approach, as local anesthesia with sedation may be associated with better outcomes in TAVR [23].

Interestingly, we found that the risk of neurovascular complications appears to be lower with the TCv approach compared to the SAo approach. This finding is particularly interesting because the risk of stroke or TIA remains a significant concern in patients undergoing TAVR procedures. It was mainly driven by the study conducted by Lederman et al. [9], which reported a rate of neurovascular complications of 13.2% at 30 days associated with the TAx approach. This rate seems unusually high when compared to previous cohort studies that investigated TAx-TAVR [24,25]. Regarding TC and TSc approaches, the rates of stroke reported in the literature usually range from 3 to 5% [22,26]. Therefore, our findings should be interpreted with caution. Another unknown factor is the proportion of patients who received cerebral embolic protection devices. While one study mentioned a very low utilization rate [9], the other two studies did not report this information [15,16].

The decision to either perform TCv-TAVR or SAo-TAVR in patients unfit for TF-TAVR is heavily contingent on the patients’ anatomical considerations. Aortic dissections, pedunculated aortic plaques, and inferior vena cava filters are relative contraindications to TCv-TAVR [27]. In contrast, contraindications to SAo approaches include small artery diameter (<6 mm), heavy calcification, excessive kinking or severe stenosis of the access vessel, prior ipsilateral carotid artery intervention, or stenosis of the contralateral carotid artery (for the TC approach).

In summary, our results suggest that TCv-TAVR may achieve outcomes comparable to SAo approaches and, from a practical standpoint, may be considered as a first-line alternative to TF-TAVR among eligible patients. The TCv access also offers additional benefits, such as avoiding the need for surgical cutdowns or thoracic incisions, as well as facilitating a rapid postoperative recovery with the utilization of large-bore venous access [16]. It remains unknown whether this may lead to shorter recovery times and hospital lengths of stay, resulting in decreased overall costs. Recently published technical recommendations should help expand its utilization in patients with contra-indications to TF-TAVR [27].

The choice between TCv-TAVR and SAo-TAVR hinges on factors such as patients’ vascular anatomy, comorbidities, and the operator’s experience. This decision should be made on a case-by-case basis, emphasizing the importance of operators’ expertise, particularly given that TCv-TAVR, while expanding in use, remains predominantly employed in high-volume and experienced centers. Transthoracic alternative vascular pathways for TAVR are now reserved by most teams for patients in whom TF, SAo, or TCv approaches are contraindicated [28].

Our study is subject to several limitations. Firstly, the absence of randomized controlled trials directly comparing TCv and SAo-TAVR restricts this meta-analysis to observational studies, which are susceptible to potential flaws. Potential confounding bias, resulting from the failure to adequately account for differences in baseline characteristics and confounding variables in the analysis, may be present. While it is theoretically possible to conduct a trial comparing the two vascular accesses in all patients contraindicated for TF-TAVR, practical obstacles such as participants’ vascular anatomy and surgeons’ expertise would pose challenges. Secondly, the authors acknowledge the scarcity of studies directly comparing TCv- to SAo-TAVR. As a result, the included patient sample size is relatively small. This limitation is attributed to the relatively recent emergence of both approaches. Additional studies are needed and will hopefully expand the available research on this comparison. Finally, the results were published by highly specialized centers boasting considerable expertise and skilled teams proficient in the use of the different vascular pathways for TAVR. The impact of this “experience” factor on the outcomes remains unknown, and the findings presented here may not be universally applicable to all teams worldwide.

## 5. Conclusions

In conclusion, among patients unfit for TF-TAVR, our meta-analysis did not find any significant difference between TCv-TAVR and SAo-TAVR regarding the rates of in-hospital or 30-day ACM and post-procedural complications. There was a potential indication of a lower risk of neurovascular complications associated with the TCv approach. These results suggest that both approaches may be considered as first-line alternatives to TF-TAVR, depending on local expertise and the patients’ anatomy and comorbidities. Furthermore, the acquisition of additional data through head-to-head comparisons and cohort studies with prolonged follow-up periods is essential. This extended investigation should encompass assessments of economic implications and the pivotal role of operator experience. Such comprehensive research efforts will give a more robust understanding of the optimal application of both SAo and TCv-TAVR in diverse clinical contexts.

## Figures and Tables

**Table 1:** Baseline characteristics of patients undergoing transcatheter aortic valve implantation.

						Comorbidities										Echocardiographic Parameters	
Authors, Year	Study Arm	Sample Size (*n*)	Age (Years)	Male Gender	STS Score	NYHA Class III or IV	HTA	Diabetes	MI	CABG	AFF	PAD	Previous Stroke/TIA	CLD/COPD	ESRD	LVEF	AV Mean Gradient (mmHg)
Paone et al. 2018 [15]	TCv	58	79.6 ± 9.6	44.8	8.0 ± 5.2	81.3	90.0	44.8	-	-	-	75.9	43.1	43.1	5.2	53.3 ± 15.5	32.0 ± 13.1
	TC	32	79.0 ± 9.6	50.0	6.9 ± 4.4	93.1	93.8	34.4	-	-	-	78.1	40.6	62.5	6.3	56.2 ± 11.9	36.5 ± 15.2
Long et al. 2020 [16]	TCv	22	80.7 ± 3.9	36.4	9.0 ± 1.9	-	-	31.8	27.3	13.6	22.7	36.4	18.1	22.7	9.0	42.3 ± 4.2	38.2 ± 4.8
	TSc	41	83.2 ± 3.7	41.5	10.4 ± 2.6	-	-	43.9	12.2	9.8	26.8	29.3	14.6	24.3	7.3	38.3 ± 5.9	34.8 ± 4.7
Lederman et al. 2022 [9]	TCv	238	76.4 ± 9.1	43.7	5.0 (3.2, 8.4)	60.1	94.1	43.9	24.4	26.9	34.6	55.3	19.8	40.3	8.8	58.0 (43.0, 60.0)	40.1 ± 13.5
	TAx	106	77.2 ± 8.8	56.6	5.6 (4.0, 8.3)	85.5	92.5	31.8	25.7	19.8	34.0	0	28.3	34.0	5.7	57.0 (43.0, 63.0)	41.1 ± 13.9

TCv = transcaval; TC = transcarotid; TSc = transsubclavian; TAx = transaxillary; STS score = Society of Surgeons score; NYHA = New York Heart Association; HTA = hypertension; MI = myocardial infarction; CABG = coronary artery by-pass graft; AFF = atrial fibrillation or flutter; PAD = peripheral artery disease; TIA = transient ischemic attack; CLD = chronic lung disease; COPD = chronic obstructive pulmonary disease; ESRD = end-stage renal disease; LVEF = left ventricular ejection fraction; AV = aortic valve. All values are expressed as percentages (%), unless specified otherwise.

## Data Availability

The original contributions presented in the study are included in the article and Supplementary Materials, further inquiries can be directed to the corresponding author.

## Supplementary Materials

The following supporting information can be downloaded at: https://www.mdpi.com/article/10.3390/jcm13020455/s1, Figure S1: Search strategy; Table S1: Newcastle-Ottawa scale, Table S2: Comparison of dichotomous baseline characteristics, Table S3: Comparison of continuous baseline characteristics, Table S4: Perioperative characteristics and Table S5: Comparison of perioperative characteristics.

## References

[1] Carroll J.D., Mack M.J., Vemulapalli S., Herrmann H.C., Gleason T.G., Hanzel G., Deeb G.M., Thourani V.H., Cohen D.J., Desai N. (2020). STS-ACC TVT Registry of Transcatheter Aortic Valve Replacement. J. Am. Coll. Cardiol..

[2] Auffret V., Lefevre T., Van Belle E., Eltchaninoff H., Iung B., Koning R., Motreff P., Leprince P., Verhoye J.P., Manigold T. (2017). Temporal Trends in Transcatheter Aortic Valve Replacement in France: FRANCE 2 to FRANCE TAVI. J. Am. Coll. Cardiol..

[3] Greenbaum A.B., O’Neill W.W., Paone G., Guerrero M.E., Wyman J.F., Cooper R.L., Lederman R.J. (2014). Caval-aortic access to allow transcatheter aortic valve replacement in otherwise ineligible patients: Initial human experience. J. Am. Coll. Cardiol..

[4] Modine T., Lemesle G., Azzaoui R., Sudre A. (2010). Aortic valve implantation with the CoreValve ReValving System via left carotid artery access: First case report. J. Thorac. Cardiovasc. Surg..

[5] Ruge H., Lange R., Bleiziffer S., Hutter A., Mazzitelli D., Will A., Schreiber C., Laborde J.C., Bauernschmitt R. (2008). First successful aortic valve implantation with the CoreValve ReValving System via right subclavian artery access: A case report. Heart Surg. Forum.

[6] De Robertis F., Asgar A., Davies S., Delahunty N., Kelleher A., Trimlett R., Mullen M., Moat N. (2009). The left axillary artery--a new approach for transcatheter aortic valve implantation. Eur. J. Cardiothorac. Surg..

[7] Overtchouk P., Modine T. (2018). Alternate Access for TAVI: Stay Clear of the Chest. Interv. Cardiol. Rev..

[8] Lu H., Monney P., Fournier S., Pavon A.G., Roguelov C., Eeckhout E., Muller O., Kirsch M. (2021). Transcervical approach versus transfemoral approach for transcatheter aortic valve replacement. Int. J. Cardiol..

[9] Lederman R.J., Babaliaros V.C., Lisko J.C., Rogers T., Mahoney P., Foerst J.R., Depta J.P., Muhammad K.I., McCabe J.M., Pop A. (2022). Transcaval Versus Transaxillary TAVR in Contemporary Practice: A Propensity-Weighted Analysis. JACC Cardiovasc. Interv..

[10] Recommendations for the Conduct, Reporting, Editing, and Publication of Scholarly Work in Medical Journals. May 2023. https://www.icmje.org/icmje-recommendations.pdf.

[11] Page M.J., McKenzie J.E., Bossuyt P.M., Boutron I., Hoffmann T.C., Mulrow C.D., Shamseer L., Tetzlaff J.M., Akl E.A., Brennan S.E. (2021). The PRISMA 2020 statement: An updated guideline for reporting systematic reviews. BMJ.

[12] Kappetein A.P., Head S.J., Généreux P., Piazza N., van Mieghem N.M., Blackstone E.H., Brott T.G., Cohen D.J., Cutlip D.E., van Es G.A. (2012). Updated standardized endpoint definitions for transcatheter aortic valve implantation: The Valve Academic Research Consortium-2 consensus document (VARC-2). Eur. J. Cardiothorac. Surg..

[13] Généreux P., Piazza N., Alu M.C., Nazif T., Hahn R.T., Pibarot P., Bax J.J., Leipsic J.A., Blanke P., VARC-3 Writing Committee (2021). Valve Academic Research Consortium 3: Updated endpoint definitions for aortic valve clinical research. Eur. Heart J..

[14] Lo C.K.L., Mertz D., Loeb M. (2014). Newcastle-Ottawa Scale: Comparing reviewers’ to authors’ assessments. BMC Med. Res. Methodol..

[15] Paone G., Eng M., Kabbani L.S., Borgi J., Peterson E., Novitsky B., Burroughs B., Wang D.D., O’Neill W.W., Greenbaum A.B. (2018). Transcatheter Aortic Valve Replacement: Comparing Transfemoral, Transcarotid, and Transcaval Access. Ann. Thorac. Surg..

[16] Long A., Mahoney P. (2020). Comparative Intermediate-Term Outcomes of Subclavian and Transcaval Access for Transcatheter Aortic Valve Replacement. J. Invasive Cardiol..

[17] Abellan C., Antiochos P., Fournier S., Skali H., Shah P., Maurizi N., Eeckhout E., Roguelov C., Monney P., Tzimas G. (2023). Extrathoracic Against Intrathoracic Vascular Accesses for Transcatheter Aortic Valve Replacement: A Systematic Review with Meta-Analysis. Am. J. Cardiol..

[18] Van der Boon R.M.A., Marcheix B., Tchetche D., Chieffo A., Van Mieghem N.M., Dumonteil N., Vahdat O., Maisano F., Serruys P.W., Kappetein A.P. (2014). Transapical versus transfemoral aortic valve implantation: A multicenter collaborative study. Ann. Thorac. Surg..

[19] Arai T., Romano M., Lefèvre T., Hovasse T., Farge A., Le Houerou D., Hayashida K., Watanabe Y., Garot P., Benamer H. (2016). Direct Comparison of Feasibility and Safety of Transfemoral Versus Transaortic Versus Transapical Transcatheter Aortic Valve Replacement. J. Am. Coll. Cardiol. Intv..

[20] Greenbaum A.B., Babaliaros V.C., Chen M.Y., Stine A.M., Rogers T., O’Neill W.W., Paone G., Thourani V.H., Muhammad K.I., Leonardi R.A. (2017). Transcaval Access and Closure for Transcatheter Aortic Valve Replacement: A Prospective Investigation. J. Am. Coll. Cardiol..

[21] Lederman R.J., Babaliaros V.C., Rogers T., Stine A.M., Chen M.Y., Muhammad K.I., Leonardi R.A., Paone G., Khan J.M., Leshnower B.G. (2019). The Fate of Transcaval Access Tracts: 12-Month Results of the Prospective NHLBI Transcaval Transcatheter Aortic Valve Replacement Study. JACC Cardiovasc. Interv..

[22] Lu H., Monney P., Hullin R., Fournier S., Roguelov C., Eeckhout E., Rubimbura V., Faroux L., Barrier A., Muller O. (2021). Transcarotid Access Versus Transfemoral Access for Transcatheter Aortic Valve Replacement: A Systematic Review and Meta-Analysis. Front. Cardiovasc. Med..

[23] Fröhlich G.M., Lansky A.J., Webb J., Roffi M., Toggweiler S., Reinthaler M., Wang D., Hutchinson N., Wendler O., Hildick-Smith D. (2014). Local versus general anesthesia for transcatheter aortic valve implantation (TAVR)—Systematic review and meta-analysis. BMC Med..

[24] Schäfer U., Deuschl F., Schofer N., Frerker C., Schmidt T., Kuck K.H., Kreidel F., Schirmer J., Mizote I., Reichenspurner H. (2017). Safety and efficacy of the percutaneous transaxillary access for transcatheter aortic valve implantation using various transcatheter heart valves in 100 consecutive patients. Int. J. Cardiol..

[25] Zhan Y., Toomey N., Ortoleva J., Kawabori M., Weintraub A., Chen F.Y. (2020). Safety and efficacy of transaxillary transcatheter aortic valve replacement using a current-generation balloon-expandable valve. J. Cardiothorac. Surg..

[26] Faroux L., Junquera L., Mohammadi S., Del Val D., Muntané-Carol G., Alperi A., Kalavrouziotis D., Dumont E., Paradis J.M., Delarochellière R. (2020). Femoral Versus Nonfemoral Subclavian/Carotid Arterial Access Route for Transcatheter Aortic Valve Replacement: A Systematic Review and Meta-Analysis. J. Am. Heart Assoc..

[27] Lederman R.J., Greenbaum A.B., Khan J.M., Bruce C.G., Babaliaros V.C., Rogers T. (2023). Transcaval Access and Closure Best Practices. JACC Cardiovasc. Interv..

[28] Lu H., Fournier S., Namasivayam J., Roguelov C., Ferrari E., Eeckhout E., Monney P., Tozzi P., Marcucci C., Muller O. (2020). Transapical approach versus transcervical approach for transcatheter aortic valve replacement: A retrospective monocentric study. Interact. Cardiovasc. Thorac. Surg..

