# Supplementary material for: Transcaval versus Supra-Aortic Vascular Accesses for Transcatheter Aortic Valve Replacement: A Systematic Review with Meta-Analysis
Source: J Clin Med. 2024 Jan 14;13(2):455. doi: 10.3390/jcm13020455 (PMC10816274; doi:10.3390/jcm13020455)
Supplement: Supplementary file 1 [file jcm-13-00455-s001.zip › jcm-2798239-supplementary.pdf]

Supplementary data

Supplementary Figure S1. Search strategy

Pubmed/Medline

(Transcatheter Aortic Valve Replacement OR transcatheter aortic valve implantation OR TAVR or TAVI) AND (transcaval OR caval-aortic OR transcarotid OR carotid OR brachiocephalic OR transsubclavian OR subclavian OR transaxillary OR axillary)

Embase

('transcatheter aortic valve replacement'/exp OR 'transcatheter aortic valve replacement' OR (transcatheter AND aortic AND ('valve'/exp OR valve) AND ('replacement'/exp OR replacement)) OR 'transcatheter aortic valve implantation'/exp OR 'transcatheter aortic valve implantation' OR (transcatheter AND aortic AND ('valve'/exp OR valve) AND ('implantation'/exp OR implantation)) OR tavr OR 'tavi'/exp OR tavi) AND (transcaval OR 'caval aortic' OR transcarotid OR 'carotid'/exp OR carotid OR brachiocephalic OR transsubclavian OR subclavian OR transaxillary OR axill ary)

Supplementary Table S1. Newcastle-Ottawa scale

Study	Study design	Selection			Comparability	Outcomes			Total score
		Representativeness of exposed cohort	Selection of non-exposed cohort	Ascertainment of exposure	Comparability of cohorts on the basis of the design or analysis	Assessment of outcomes	Length of follow-up of cohorts	Adequacy of follow-up of cohorts	
Paone et al. 2018 (15)	restrospective monocentric cohort	*	*	*	*	0	*	*	6/8
Long et al. 2020 (16)	restrospective monocentric cohort	*	*	*	*	0	*	*	6/8
Lederman et al. 2022 (9)	restrospective multicentric cohort	*	*	*	*	*	*	*	7/8

The lack of star for the assessment of outcomes for Paone et al. and Long et al. is justified by the fact that the Valve Academic Research Consortium criteria (either 2 or 3) were not used.

Supplementary Table S2. Comparison of dichotomous baseline characteristics

Characteristics	Number of studies (references)	TCv		SAo		p value
		n	%	n	%	
Male gender	3 (9, 15, 16)	138	43.3	93	52.0	0.07
HTA	2 (9, 15)	275	92.9	128	92.8	0.95
Diabetes	3 (9, 15, 16)	123	38.7	75	41.9	0.48
Prior MI	2 (9, 16)	64	24.6	32	21.8	0.52
CABG	2 (9, 16)	67	25.8	25	17.0	0.04
AFF	2 (9, 16)	87	33.5	47	32.0	0.76
PAD	2 (15, 16)	52	65.0	37	50.7	0.07
Previous stroke/TIA	3 (9, 15, 16)	81	25.5	45	25.1	0.93
CLD/COPD	3 (9, 15, 16)	126	39.6	65	36.3	0.47
ESRD	3 (9, 15, 16)	26	8.2	11	6.1	0.41

TCV = transcaval; SAo = supra-aortic; HTA = hypertension; MI = myocardial infarction; CABG = coronary artery by-pass graft; AFF = atrial fibrillation or flutter; PAD = peripheral artery disease; TIA = transient ischemic attack; CLD = chronic lung disease; COPD = chronic obstructive pulmonary disease; ESRD = end-stage renal disease.

Supplementary Table S3**.** Comparison of continuous baseline characteristics

Variables	Number of studies (references)	TCv	SAo	Mean difference (95%)	p-value
Age (years)	3 (9, 15, 16)	77.3 ± 9.0	78.9 ± 8.4	-1.4 (-2.8, -0.1)	0.04
STS score	2 (15, 16)	8.3 ± 4.6	8.9 ± 3.9	-0.8 (-1.8, 0.2)	0.11

TCv = transcaval; SAo = supra-aortic; STS score = Society of Thoracic Surgeons.

Supplementary Table S4. Perioperative characteristics

Study	Study arm	Sample size (n)	Moderate sedation	Procedural success	BE type of valve
Paone et al. 2018 (15)	TCv	58	-	-	82.8
	TC	32	0	-	93.8
Long et al. 2020 (16)	TCv	22	64.6	100.0	45.4
	TSc	41	19.5	100.0	33.1
Lederman et al. 2022 (9)	TCv	238	45.4	99.2	-
	TAx	106	14.2	99.1	-

TCv = transcaval; TC = transcarotid; TSc = transsubclavian; TAx = transaxillary; BE = balloon-expendable. All values are expressed as percentages (%), unless specified otherwise.

Supplementary Table S5. Comparison of perioperative characteristics

Characteristics	Number of studies (references)	TCv		SAo		p value
		n	%	n	%	
Moderate sedation	3 (9,15,16)*	122	46.9	23	12.8	< 0.001
Procedural success	2 (9,16)	257	98.8	146	99.3	0.64
BE type of valve	2 (15,16)	58	72.5	44	60.3	0.11

TCv = transcaval; SAo = supra-aortic; BE = balloon-expendable. All values are expressed as percentages (%), unless specified otherwise. *Information on anesthesia modality in patients undergoing TCv procedures not available in Paone et al.
